# Wild Italian *Hyssopus officinalis* subsp. *aristatus* (Godr.) Nyman: From Morphological and Phytochemical Evidences to Biological Activities

**DOI:** 10.3390/plants10040631

**Published:** 2021-03-26

**Authors:** Alessandra Guerrini, Gianni Sacchetti, Monica Paulina Echeverria Guevara, Guglielmo Paganetto, Alessandro Grandini, Immacolata Maresca, Luigi Menghini, Luciano Di Martino, Arianna Marengo, Massimo Tacchini

**Affiliations:** 1 Pharmaceutical Biology Lab., Research Unit 7 of Terra&Acqua Tech Technopole Lab., Department of Life Sciences and Biotechnology, University of Ferrara, Piazzale Luciano Chiappini, 3, 44123 Ferrara, Italy; alessandra.guerrini@unife.it (A.G.); gianni.sacchetti@unife.it (G.S.); pgngll@unife.it (G.P.); grnlsn1@unife.it (A.G.); mci@unife.it (I.M.); 2 Department of Earth Science, Universidad Estatal Amazónica, Puyo 160106, Ecuador; mecheverria@uea.edu.ec; 3 Department of Pharmacy, Botanical Garden “Giardino dei Semplici”, Università degli Studi “Gabriele d’Annunzio”, Via dei Vestini 31, 66100 Chieti, Italy; luigi.menghini@unich.it; 4 Ufficio Monitoraggio e Conservazione Biodiversità Vegetale, Ente Parco Nazionale della Majella, Via Badia, 28 loc. Badia Morronese, 67039 Sulmona (AQ), Italy; luciano.dimartino@parcomajella.it; 5 Department of Drug Science and Technology, University of Torino, Via P. Giuria 9, 10125 Torino, Italy; arianna.marengo@unito.it

**Keywords:** *Hyssopus officinalis* subsp. *aristatus*, macro and microscopic analysis, essential oils, ethanolic extract, chemical characterization, antioxidant, cytotoxic potential

## Abstract

Three specimens of *H. officinalis* subsp. *aristatus* were collected in three areas of the Abruzzo region (Italy) and subjected to macroscopic and microscopic observation to support their botanical identification. The essential oils (EOs) obtained from the aerial parts of the samples were characterized with the object to define their phytochemical and pharmaceutical biology profile. They highlight three different chemotypes, including one never seen in previous literature (CIV17-EO, distilled from sample harvested in 2017 at Civitaretenga), that showed a fingerprinting with the predominance of (-)-limonen-10-yl-acetate (67.9%). In 2017 European Food Safety Authority (EFSA) reported the genotoxicity of similar compounds, therefore, to dismiss any safety concern for the CIV17-EO use as flavouring substance, the Ames test was performed with no evidence of mutagenic activity. Safety of use coupled with chemical characterization of this new chemotype set the stage for a better standardization of *H. officinalis* EOs. The ethanolic extracts, on the other hand, with qualitatively similar chemical profiles in which caftaric, chlorogenic and rosmarinic acid were the main molecules, showed interesting antioxidant activity and a slight cytotoxicity towards the A549 cell line that could indicate a starting point for the evaluation of an additional preventive tool for maintaining health status.

## 1. Introduction

The genus *Hyssopus* (Lamiaceae) is represented by almost 70 species [1] but only seven are accepted as systematically ascertained, including *Hyssopus officinalis* L. that comprises four accepted subspecies that are *H. officinalis* subsp. *aristatus* (Godr.) Nyman, *H. officinalis* subsp. *canescens* (DC.) Nyman, *H. officinalis* subsp. *montanus* (Jord. & Fourr.) Briq., *H. officinalis* subsp. *officinalis* L. All other *Hyssopus* species, subspecies and varieties are considered to be synonyms [1,2,3]. *H. officinalis* is a perennial and semi-perennial aromatic plant with a pungent pleasant smell, growing wild in dry, rocky, or almost sandy or silty, calcareous soils and in Mediterranean like climatic conditions [4,5]. The species have an herbaceous, shrubby, or sub-shrubby growth habit and grows up to 0.2–0.6 m in height. The stems are straight, sometimes woody in higher parts, and branched at the base. The leaves are opposite, lanceolate (2–4 cm long and 2–7 mm wide), generally revolute, with surfaces covered by glandular trichomes. The flowers are hermaphrodite and can be blue, pink, pale violet, white or purple in color. The ripe fruit is small, oblong, dihedral shaped, dark brown, and up to 25 mm in length. The natural area of *H. officinalis* is widespread in Africa, Asia, and Europe, but the species is also cultivated in Asia, China, Central and Southern Europe, and in North America both by generative and vegetative propagation [6,7,8,9].

The parts used (crude drug), both from wild and cultivated plants, are the aerial parts (Herba hissopi) known by traditional medicine to have health properties to treat several digestive, genitourinary, and respiratory disorders [3,6,10]. *H. officinalis* and its subspecies are also known to be used as cooking spices, mainly thanks to the volatile fraction responsible of the aromatic properties of the crude material, today appreciated also as cosmetic ingredient for its warm and typical fragrance. In fact, the essential oil obtained through steam distillation from *H. officinalis* and its subspecies, is characterized for the most part by volatile oxygenated monoterpene and monoterpene hydrocarbons, followed by minor sesquiterpene hydrocarbons and phenolic fractions. Other important compounds are flavonoids and free phenolic acids. The flavonoid content is mainly characterized by apigenin, luteolin, diosmin and acacetin derivatives, while the free phenolic acids fraction mainly by chlorogenic, ferulic, syringic, caffeic, *p*-hydroxybenzoic, catechuic, vanillic, *p*-coumaric, rosmarinic and genistic acids [3,6,10].

The chemical profile of plants is known to reflect the resilience of plants within the same species due to different climatic conditions, date of collections, allelopathy and many other environmental conditions and *H. officinalis* with its subspecies clearly reflects this aspect. For what concerns *Hyssopus* essential oils, that we may consider as the paradigmatic subject of the relationship between plant resilience and plant phytochemistry, there are strong compositional differences among the subspecies and between plants belonging to the same subspecies as clearly pointed out by literature and despite ISO 9841 directive [3,6,11]. For example, *H. officinalis* subsp. *aristatus* collected in different areas characterized by different environmental and climatic conditions and in different years, revealed important differences in essential oil composition [6,12]. For what concerns the phenolic fraction, the differences where less evident and seemed regard mainly the relative compounds proportions, in particular between chlorogenic acid, 4-O-caffeoylquinic acid, 5-O-caffeoylquinic acid and 4-O-feruloylquinic acid [12]. As a consequence of this chemodiversity, the bioactivity of essential oils and of the phenolic fraction may significantly vary, affecting the standardization of efficacy and safety of the crude drug and its phytocomplexes, as evidenced by related literature regarding in vitro and in vivo studies, mainly reflecting antimicrobial, antioxidant, anti-viral, immunomodulatory, spasmolytic, platelet aggregation properties, the most relevant being [3,6,10]. In light of these premises, *Hyssopus officinalis* subsp. *aristatus* (Godr.) Nyman represents a valuable research subject to clarify these variability aspects.

The present study points out the morphological, phytochemical and bioactivity aspects of *H. officinalis* subsp. *aristatus* samples collected in different years in three representative locations in the Abruzzo Region (Central Italy) during the balsamic period. A pharmaceutical biology and pharmacognostic approach have been applied for morphological characterization of the samples to point out any possible significant morphological differences between the samples collected in different years. Similarly, the crude drugs have been subjected to standardized extraction procedures to obtain EOs and phenolic extracts, that have been chemically characterized and subjected to in vitro biological assays to point out differences among the harvested samples. The research would give a contribute to the recent publications regarding *H. officinalis* subsp. *aristatus*, shedding light on a new EO chemotype, to improve the standardization of herbal products based on this extract, while also evaluating its safety and efficacy.

Cancer is one of the most important health problems of modernity, and natural compounds are considered more and more valuable candidates for the development of new intervention strategies to improve the low therapeutic index and the frequent occurrence of chemoresistance of the current anticancer therapies. The initial stages of carcinogenesis could be suppressed by antioxidants from plant sources through several modalities, for this reason the evaluation of antioxidant activity of *H. officinalis* subsp. *aristatus* was accompanied by an assessment, albeit preliminary, of chemopreventive activity. These evaluations have been supported by in silico studies, used to obtain preliminary information on the absorption and distribution, after oral intake, of secondary metabolites present in the extracts in order to evaluate if the effects obtained in vitro can be hypothesized also in vivo. Therefore, this approach aims to take the first steps toward an assessment of in vitro biological activity that could exhibit an in vivo correspondence.

## 2. Results and Discussion

### 2.1. Macroscopic and Microscopic Analysis

The specimen of *H. officinalis* subsp. *aristatus*, collected in 2017 at Civitaretenga (Italy), was subjected to stereomicroscopic and optical microscopic observation to authenticate the main characteristics described in literature for the subspecies and to identify any other diagnostic elements supporting botanical recognition [13]. The specimens collected at Campo di Giove and Navelli (Italy) in 2019 presented similar microscopic and pharmacognostic characters already observed for the first one.

The plant material consisted of the aerial parts (stems, leaves, calyces, flowers, fruits). The bilabial corolla was blue-violet, the other remaining parts were brownish-green. The odor was aromatic, typical of Lamiaceae family.

**Figure 1:**
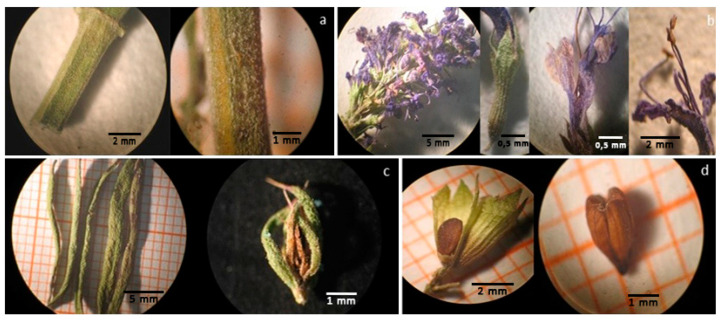
Morphological traits of *H. officinalis* subsp. *aristatus*. (**a**) The stem is quadrangular and pale green; (**b**) Flowers grouped in axillary verticillaster, facing one side—calyx slightly reddened, pubescent, tubular, and with aristiform teeth—flower with bilobo upper lip—flower in which long protruding stamens can be seen; (**c**) leaves lanceolate, briefly petiolate—acute floral leaves, aristate at the apex; (**d**) ovoid or oblong tetrachene, with three obtuse edges, glabrous and with minute dimples (fruit).

The observation at the stereomicroscope highlighted the following morphological traits: the woody quadrangular stems with tiny trichomes (0.1 mm, Figure 1a); the spike inflorescences with unilateral clusters of flowers, having synsepalous and conical calyx with aristate teeth, gamopetalous corolla, blue-violet in color, with a bilobed upper lip and a trilobed lower lip and 4 long protruding stamens (Figure 1b); the 20–30 mm long leaves, revolute at the margins and floral leaves with an aristate tip, characteristic of this subspecies (Figure 1c); the fruit with four one-seeded nutlets of about 1–2 mm contained within the calyx also typical of Lamiaceae family (Figure 1d).

**Figure 2:**
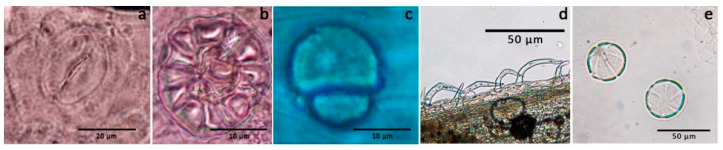
Microscopic examination of *H. officinalis* subsp. *aristatus*. (**a**) Diacitic stomata; (**b**) secretory hairs with an octocellular head; (**c**) secretory hairs with a bicellular head; (**d**) stubby, hook-shaped unicellular covering hairs; (**e**) pollen grains with slightly granular, six-slit exine.

The microscopic examination showed the following diagnostic characters: diacitic stomata in leaves and calyces (Figure 2a); glandular trichomes in the flower, consisting of 8 cells and 2 cells (Figure 2b,c) and covering hooked trichomes on the surface of the stems; pollen in the flower.

The microscopic features are in accordance with previously reported data [6], while the description of the stomata apparatus had not yet been given.

### 2.2. Extraction and Chemical Characterization of H. officinalis subsp. aristatus Essential Oils 

The essential oils extracted by hydrodistillation from the aerial parts of *H. officinalis* subsp. *aristatus* were light yellow and showed a yield from 0.28 to 0.70% (Table 1), similar to that reported by other authors respectively of 0.6–1.1% by Venditti et al. [6], 0.24–2.0% by Hajdari et al. [12], 0.6% by Džamić et al. [14].

The two samples collected in 2019 highlighted a chemical composition of essential oil typical of the two chemotypes already described in literature [12,16], characterized by the prevalence of methyleugenol (41.5%), 1,8-cineole (39.7%) and limonene (7.6%) for the Campo di Giove one (CdG19-EO) and *cis*-pinocamphone (43.2%), methyleugenol (15.8%), *trans*-pinocamphone (11.0%) and 1,8-cineole (4.4%) for the other collected at Navelli (NAV19-EO).

Moreover, considering other literature data [4,6,14] methyleugenol, 1,8-cineole, *cis*-pinocamphone, *trans*-pinocamphone and β-pinene can be considered the main compounds of *H. officinalis* subsp. *aristatus* essential oil.

**Figure 3:**
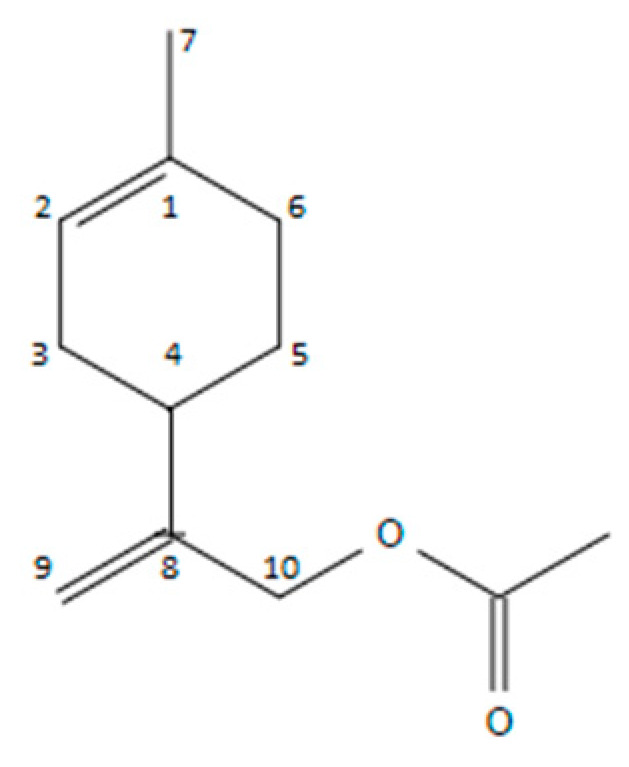
(-)-Limonen-10-yl-acetate (*p*-mentha-1,8-dien-10-yl acetate).

On the basis of our results, there was instead a substantial difference in the composition of CIV17-EO when compared to the previous chemotypes. It showed a fingerprinting with the predominance of (-)-limonen-10-yl-acetate (67.9%) (Figure 3), which is not detectable in the previous literature data, followed by 1,8-cineole (15.5%), limonene (5.8%), limonen-10-ol (2.8%), limonen-10-ol methyl ether (1.9%), β-pinene (1.7%) and germacrene D (1.2%). It was therefore composed of a greater content of monoterpenes, deriving from limonene and a 2% of sesquiterpenes. To date, no authors has described this particular chemical composition for the essential oil of this subspecies. 

Limonen-10-yl-acetate is found to be an Food and Drug Administration (FDA)-approved flavoring substance, identified in mandarins, lemons, peppermint and grapefruit. In the European Union, however, it has been removed from the list of authorized flavorings by European Food Safety Authority (EFSA) [17], since it is suspected of genotoxicity.

This possible new chemotype could be explained with a peculiar biodiversity of the specific area, but we will perform further analyses on plants collected in the same area in different years to support this hypotesis.

The structure of the main component (67.9%) of the essential oil was not elucidated with mass spectrum alone, but also with NMR. The mass spectrum had a molecular ion (*m/z* 134) which differed from limonen-10-ol (*m/z* 152) by the loss of water (*m/z* 18).

Its ^1^H-NMR spectrum compared with the limonene one showed: (1) a methylene group with protons that had a chemical shift of 4.95 and 5.05 ppm instead of the methyl group of the limonene isopropenyl at the 1.70 ppm, (2) a methyl group with a typical acetate chemical shift at 2.07 ppm, (3) vinyl protons with a shifted signal, due to the presence of the acetate group, at 4.58 ppm instead of the typical one in limonene at 4.70 ppm. Similarly, the chemical shifts of ^13^C-NMR spectrum were assigned comparing with the limonene one [18].

The characterization data for this molecule was as follows: (-)-limonen-10-yl-acetate. (GC: 96%, Chloroform, [α]^2^^5^_D_ -46.8), ^1^H-NMR (CDCl_3_, 400 MHz): δ ppm 5.40 (1H, m, H-2), 5.05–4.95 (2H, s, H-10_a,b_), 5.58 (2H, s, H-9), 2.07 (3H, s, acetyl group), 2.20–1.80 (7H, m, H-3,4,5,6), 1.65 (3H, s, H-7). ^13^C-NMR (CDCl_3_, 100 MHz): δ 170.8 (CO), 148.3 (C-8), 133.8 (C-1), 120.3 (C-2), 111.0 (C-9), 66.2 (C-10), 37.0 (C-3″), 76.40 (C-4), 31.0 (C-6), 30.4 (C-3), 27.9 (C-5), 23.4 (C-7), 21.0 (CH_3_-acetyl).

The structure of limonen-10-ol methyl ether was deduced by comparing the fragmentation pattern to that of limonen-10-ol and observing the following differences: the molecular ion (*m/z* 166) lost a methyl radical (*m/z* 15) to give the cation ion (*m/z* 151) corresponding to limonen-10-ol one. Moreover, the shift of arithmetic index of a couple of compounds, thymol and thymol methyl ether (AI: 1289 and 1232, respectively) [15], was comparable to that of limonen-10-ol and limonen-10-ol methyl ether (exp.AI: 1291 and 1235, respectively; AI: 1288 for limonen-10-ol from literature [15].

### 2.3. Chemical Characterization of H. officinalis subsp. aristatus Ethanolic Extracts

The 70% ethanolic extracts was performed in duplicate for all samples and the yields in terms of dry weight were calculated. The Civitaretenga ethanolic extract (CIV17-UAE) exhibited the best yield: 13.0 ± 0.8%.

All phytocomplexes showed qualitatively similar profiles confirming the presence in all samples of caftaric acid (UV: λ_max_ = 327 nm; [M-H]^−^: *m/z* 311), chlorogenic acid (UV: λ_max_ = 327 nm; [M-H]^−^: *m/z* 353) and rosmarinic acid (UV: λ_max_ = 327 nm; [M-H]^−^: *m/z* 359).

The quantitative RP-HPLC-DAD analyses showed a clear prevalence of chlorogenic acid respect to the other metabolites identified in all the extracts, for the two years and the three collection areas. Furthermore, the CIV17-UAE showed a higher concentration of all the molecules respect to those of 2019 (NAV19-UAE and CdG19-UAE), even with a higher yield (Table 2).

### 2.4. Antioxidant Activity of H. officinalis subsp. aristatus

Plants growing under unfavorable conditions generate high concentrations of reactive oxygen species (ROS), which can cause oxidative stress. To prevent this, cells bring into play enzymatic and non-enzymatic elements that constitute the complex plant antioxidant system [19]. The non-enzymatic part of this important system consists of molecules (e.g., phenols) that have various mechanisms of action, such as the inhibition of enzymes, the chelation of trace elements involved in the production of free radicals, or the absorption of reactive species.

This study focuses on the radical scavenging activity of ethanolic extracts and EOs obtained from three samples of *H. officinalis* subsp. *aristatus* tested with DPPH and ABTS assay. In addition to the extracts, this study considered some standard molecules identified in the phytocomplex (chlorogenic acid, caftaric acid and rosmarinic acid), a synthetic antioxidant (trolox) and thymol (example of active terpene); the latter two used as positive controls (Table 3).

The essential oils CIV17-EO and NAV19-EO did not possess any antioxidant capacity, while CdG19-EO showed weak activity. The latter EO consists mainly of methyleugenol (41.5%), 1,8 cineole (39.7%) and limonene (7.6%), a composition which could explain the higher antioxidant activity compared to the other [20,21].

The EtOH extracts, on the other hand, showed interesting antioxidant activity, although with IC_50_ values an order of magnitude lower than the positive control and the pure molecules identified in the various extracts. On the other hand, all extracts are active at 50 μg/mL or less, a concentration at which no cytotoxic activity was observed on human keratinocytes (HaCat cell line; Table 4).

Considering now the relationship between the bioactivity and the chemical composition of the various extracts, especially from a quantitative point of view, it is observed that extract CIV17-UAE is the richest of the three characterized compounds, and the one that showed the best antioxidant activity.

It has been shown that the initial stages of carcinogenesis are suppressed by antioxidants from plant sources, e.g., dietary polyphenols [22]. Compounds belonging to this molecular category exert anti-cancer effects through several modes of action including alterations in cell signaling changes in cell cycle progression and modulation of enzymatic activities [23]. Based on the evidence of the antioxidant capacities the effects of extracts were further tested on a human cancer cell line.

### 2.5. Cytotoxic Activity of H. officinalis subsp. aristatus

Cancer is one of the most important health problems of modernity. In particular, the World Health Organization (WHO) indicate the lung cancer as the most common type of cancer in both men and women with 2.09 million cases in 2019 [24,25]. The transformation of normal cells into cancerous cells is a complex process regulated at every stage by a multitude of factors, each of which can be a target for anticancer agents [26]. The path of prevention could be the key to improve this global situation and could be based on healthy habits that could ward off the risk of developing cancer, such as a healthy diet [27]. This approach aims at hitting several targets together, and in this context, plants naturally follow precisely the multi-target approach to life due to their resilient nature, and due to the fact that they need to defend themselves against a multitude of pests and predators without the ability to move. Their secondary metabolites, exhibiting various mechanisms of action, can play a key role in cancer prevention. Moreover, the idea that the pharmacological action of a phytocomplex is due to a single compound is now almost definitively abandoned. In its place, the belief that plant extracts can rely on additive and synergistic effects between their constituents for their pharmacological action has taken hold. They can perform their activity in concert, and this may involve the protection of an active substance from degradation by enzymes, modification of transport across barriers, circumvention of multi-drug resistance mechanisms or other signals to the host’s cell that results in a changed efficacy of the botanical drug when compared with isolated compounds [28]. For this reason, the plant kingdom is being extensively investigated in search of possible preventive or anti-cancer agents. The literature contains numerous examples of plant extracts with anticancer activity [29,30], and in particular the Lamiaceae family could represent an effective source of active compounds against cancer cell lines [31].

Therefore, considering that *Hyssopus officinalis* subsp. *aristatus* is part of the Lamiaceae family, that is used in traditional medicine to treat respiratory disorders and that could be also introduced into the diet because its volatile fraction makes it a popular cooking spice, the aim of these tests was to verify the possible activity of the hydroalcoholic extracts and the different chemotypes of EOs against a lung cancer cell line (A549).

All extracts of *H. officinalis* were subjected to the MTT assay [32] for the evaluation of their cytotoxic effects on human keratinocyte (HaCaT) and lung adenocarcinoma (A549) during 72 h of exposure. The obtained data were compared with the values of the medium with DMSO 0.1% (negative control), and doxorubicin was used as positive control.

HaCat cell line incubated in presence of increasing concentrations of EOs (10, 20, 50, 100, 150, 200 μg/mL) showed no significant differences compared to the negative control (*p* > 0.05), indicating lack of toxicity towards the considered cell line. This result, i.e., the lack of cytotoxicity of the EOs on the HaCat cell line, underlines their safety for possible health-care/cosmetic use regardless of the chemotype considered. 

Tests performed on the A549 line (Table 4) showed a different output: all EOs exhibited a mild cytotoxicity, suggesting a partial selectivity of action of these phytocomplexes towards neoplastic cells.

**Figure 4:**
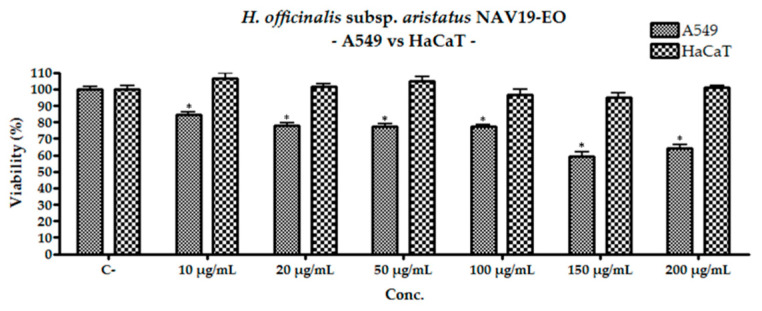
Comparison of the cytotoxic activity of NAV19-EO (location “Navelli”) against A549 and HaCat cell lines; the data presented are the result of the average of three repeated experiments ± the standard deviation; * indicate the significance of the results (*p* < 0.01) compared to the untreated cells.

CIV17-EO showed cytotoxicity at the concentration of 200 μg/mL decreasing the cell viability by about 12% compared to the negative control (*p* < 0. 01). The extract CdG19-EO showed a higher cytotoxicity (about 20%) than the previous sample. Finally, the essential oil NAV19-EO exhibited the highest cytotoxicity among the EOs (*p* > 0.01) showing a decrease in cell viability since the lowest concentration tested, without however leading to a decrease of viability of 50% and therefore to the possibility to calculate an IC_50_ value. Comparing the action of the latter extract on the two cell lines considered (Figure 4), the greater selectivity of action of this phytocomplex on neoplastic cells compared to healthy ones is underlined.

Terpenes have been shown to possess antitumoral activity by various mechanisms [30,33] and the NAV19-EO was chemically characterized by the 70% of monoterpene and oxygenated monoterpenoids of which about 11% are molecules known for their cytotoxic (e.g., sabinene, 1,8-cineole, terpinen-4-ol), antiproliferative (e.g., *cis*- and *trans*-thujone, *p*-cymene) and apoptosis-inducing (e.g., α-pinene, *cis*- and *trans*-thujone) activities. Moreover, this phytocomplex also contains methyl eugenol (15.8%) that is used as molecular scaffolding for the pharmacophore modeling of breast cancer invasion inhibitors [34] and for the synthesis of new derivatives with anticancer potential [35]. Therefore, the activity of this phytocomplex, although slight, could be motivated by the presence of the molecules listed above, but also by the synergies that can be created among them. Moreover, when ingested in liquid form, the T_max_ (predicted times for phytochemicals to reach maximum plasma concentration) of EOs phytochemicals ranged from 0.8–1.2 h (Figure S1) for all essential oils with predominant peaks and the prevalent phytochemicals are re-distributed without being swept away. Therefore, according to this simple model, we can assume the temporal conservation of the relative fraction composition after ingestion.

Considering the ethanolic extracts, the results exhibited a significant dose-dependent toxicity against A549 starting from the concentrations of 50 µg/mL and gradually increase at higher concentrations (Table 4).

Among all ethanolic extracts, the one obtained from the sample CIV17-UAE did not show enough toxicity to calculate an IC_50_ against the adenocarcinomic human alveolar basal epithelial cell line. Unlike the latter extract, the other two showed a slightly higher activity exhibiting IC_50_ values: NAV19-UAE showed an IC_50_ value of 148.15 ± 5.97 μg/mL, while the extract CdG19-UAE showed an IC_50_ value of 97.30 ± 3.08 μg/mL. These two samples also had cytotoxic effects on HaCat cell line, for concentration above 100 μg/mL, although without ever reaching 50% inhibition of viability (Table 4). 

**Figure 5:**
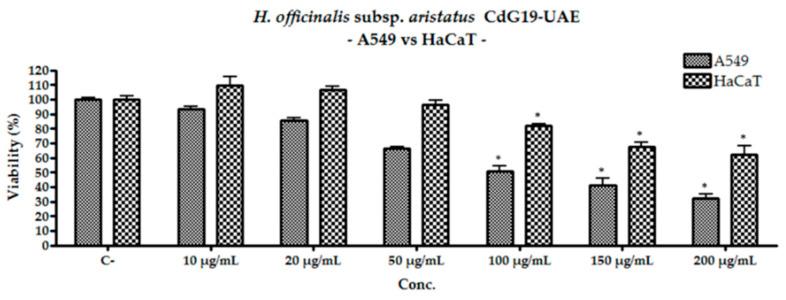
Comparison of the cytotoxic activity of ethanolic extract CdG19-UAE against A549 and HaCat cell lines; the data presented are the result of the average of three repeated experiments ± the standard deviation; * indicate the significance of the results (*p* < 0.01) compared to the untreated cells.

However, the cytotoxicity of the hydroalcoholic extracts towards this cell line is lower than that expressed on lung adenocarcinoma cells (Figure 5). Tests carried out with the sample CdG19-UAE showed that even at the highest concentration tested, the reduction in HaCat viability was approximately half that shown by A549.

As pointed out at the beginning of this section regarding the importance of multi-target therapy, in this case we have obtained the first evidence of the activity of these phytocomplexes towards the A549 cell line, that is probably due to the presence of the molecules identified, such as chlorogenic [36] and rosmarinic acid [37], and their probably synergistic activity.

To confirm these observations, the Pk-Sim PBPK model simulation for oral administration of one gram of the three ethanolic extracts, showed a substantial reduction of the quantified compounds in lung intracellular, interstitial and plasma concentration compared to the effective concentrations tested in the MTT assay, but the relative amounts of the three molecules are conserved without a substantial variation in the concentration ratios of the three components in the ethanolic extracts. PK-Sim simulation modeled on a healthy human subject 30th years old, with 73 Kg body weight and Body Mass Index of about 24 Kg/m^2^ for an oral intake of comparable dose of caftaric, chlorogenic and rosmarinic acid in ethanolic extract, shows a substantial reduction of one order of magnitude in the lung interstitial concentration when compared to the administered doses. These results must be evaluated by considering that the in vitro concentration simulate interstitial concentrations, and that effective doses in MTT test are of one order of magnitude higher of the interstitial concentrations. Nevertheless, it should be considered that simulations are performed regardless drugs reciprocal interactions, as additives, synergistic, or inhibitory effects mediated by hepatic, renal, intestinal metabolism, or drug efflux protein. Kinetic parameters related to these effects are till now not available it will be the challenger for future implementations.

### 2.6. Ames Test of H. officinalis subsp. aristatus

We tested the essential oil obtained from the sample collected on 09/2017 in the Civitaretenga area because it consists of an unpublished chemotype characterized by limonen-10-yl-acetate (*p*-mentha-1,8-dien-10-yl acetate). EFSA, in the report “Scientific Opinion on Flavouring Group Evaluation 208Revision 2 (FGE.208Rev2): Consideration of genotoxicity data on alicyclic aldehydes with α,β-unsaturation in ring/side-chain and precursors from chemical 2.2 of FGE.19” [38], concluded that *p*-mentha-1,8-dien-7-al is genotoxic in vivo and, therefore there is a safety concern for its use as flavouring substance.

Mutagenic activity was tested using the widespread and consolidated Ames test to verify the genotoxic safety of *H. officinalis* subsp. *aristatus* essential oil. There are numerous strains of salmonella produced for mutagenic activity tests, each possessing a different mechanism and sensitivity. We have decided to test the sample on two different salmonella strains that can detect almost all potential mutagens; among the most stable strains and most commonly used in this type of test there are TA98, TA100. The TA98 strain is useful for detecting mutations that cause frameshifts, deletions or genomic insertions, it only changes the mutation site, which makes them complementary and most sensitive to some substances rather than others. TA100 mainly detects substances that cause base-pair replacement and have a different genome repair system. The results (Table 5) showed that the tested essential oil was not potentially mutagenic.

## 3. Materials and Methods

### 3.1. Plant Material

The aerial parts of *H. officinalis* subsp. *aristatus* were collected from wild population of plants at early flowering stage in proximity of Gran Sasso Massif, at Civitaretenga (L’Aquila, Italy, GPS coordinate: 42.248534, 13.713459; 800 m asl), in September 2017, and at Navelli (L’Aquila, Italy, GPS coordinate: 42.245255, 13.733620; 720 m asl) and 2019 (sample CIV17 and NAV19, respectively) and on Majella Massif, at Campo di Giove (L’Aquila, Italy, GPS coordinates 42.005993, 14.030021, 1450 m asl) in October 2019 (sample CdG19). Plant material collection were carefully performed in order to not damage the wild population and obtaining a representative sample from no less than 10 plants. The samples authentication was performed by prof. Luigi Menghini according to Pignatti [13] and voucher specimens for each sample were deposited in the Herbarium of Giardino dei Semplici (Chieti, Italy). Plant material, represented by stems, leaves and flowers, was dried in ventilated oven at 40 °C. Reached a constant weight, the samples were transferred in vacuo plastic bag and stored in the dark at room temperature until used for ultrasound assisted extraction (UAE) or hydrodistillation to obtain the essential oil (EO).

### 3.2. Macro- and Microscopic Analysis

Through visualization at stereomicroscope (SFX series, Optika, Ponteranica, Bergamo, Italy) and at upright light microscope (Primo Star, Zeiss, Castiglione Olona, Italy) macroscopic and microscopic diagnostic features were determined at University of Ferrara by dr. Immacolata Maresca.

### 3.3. Preparation of Extracts

All samples of *H. officinalis* subsp. *aristatus* dry aerial parts were milled through a 2 mm sieving ring of a Variable Speed Rotor Mill (Fritsch, Idar- Oberstein, Germany). Afterwards, 5 g of each milled plant material was added to 35 mL of ethanol and 15 mL of distilled water (drug/solvent ratio of 1:10) to obtain 70% ethanolic solution. The extractions were performed by an ultrasound device (Branson Bransonic CPXH Digital Bath 3800F, Emerson, St. Louis, MO, USA) for 30 min at room temperature. After centrifugation the 70% ethanolic extract was reduced in volume with a rotary evaporator (RV 10 digital, IKA^®^-Werke GmbH & CO. KG, Staufen im Breisgau, Germany), then lyophilized to eliminate residual water. To produce the essential oil of *H. officinalis* subsp. *aristatus*, 25 g of each milled plant material was weighed in a 1000 mL flask, then 300 mL of distilled water was added. The hydrodistillation was performed through a Clevenger type equipment. The distillation time was 3 h. At the end of the extraction process, the essential oils were stored in the dark at −18 °C.

### 3.4. GC-MS and GC-FID Analyses

The GC-MS technique was used to qualitatively analyze the essential oils of *H. officinalis* subsp. *aristatus*, the GC-FID to obtain quantitative data. The GC-MS analysis was performed with a Varian 3800 chromatograph (Varian, Palo Alto, CA, USA) equipped with a Varian Factor Four VF-5ms column (5%-phenyl-95%-dimethylpolysiloxane, internal diameter: 0.25 mm, length: 30 m) interconnected with a Varian mass spectrometer SATURN MS-4000, with electronic impact ionization, ion trap analyzer and software provided with the NIST database for the identification of components. The experimental conditions used were the following: helium carrier gas (1 mL/min), split ratio of 1:50, ionization energy (EI) 70 eV, emission current of 10 μA, the scan rate of 1 scan/s, mass range 40–400 Da.

The initial oven temperature was 55 °C, then increased to 100 °C at a rate of 1 °C/min, successively to 250 °C at a rate of 5 °C/min and finally constant at 250 °C for 15 min. The analysis was carried out by introducing 1 μL of a solution consisting of 10 μL of pure essential oil dissolved in 1mL of methylene chloride in the gas chromatograph injector. The acquisition time was 90 min. 

The experimental arithmetic index (AI) of each component was determined adding a C_8_-C_32_ n-alkanes mixture (Sigma-Aldrich Italy, Milano, Italy) to the essential oil before injection in the GC-MS equipment and analyzing it under the same conditions reported above. The identification of compounds was performed by comparing their AI_s_ and the MS fragmentation pattern with those of pure compounds, of mass spectra libraries and of literature data.

Operating conditions for GC-FID (ThermoQuest GC-Trace gas-chromatograph (ThermoQuest Italia, Rodano, Italy) were the following: injector temperature 280 °C, carrier (Helium) flow rate of 1 mL/min, and split ratio 1/50, FID temperature 250 °C. The GC-FID analysis was performed in the same conditions above described. The oil percentage composition was calculated by the normalization method from the GC peak areas, without using correction factors [15,32].

### 3.5. HPLC-DAD-MS Analysis

HPLC analyses of ethanolic extracts of *H. officinalis* subsp. *aristatus* were performed using a model PU 2089 modular HPLC system (JASCO, Tokyo, Japan,) coupled to a diode array apparatus (MD 2010 Plus) and a FinniganMAT LCQ (ThermoQuest Corp./FinniganMAT; San Jose, CA, USA) mass spectrometer module linked to an injection valve with a 20 μL sampler, according to previously described [39]. A Kinetex-C18 column (150×4.6 mm, 100 Å) was used. The method of quantification was validated and parameters are the following: for caftaric acid the calibration range was 2.5–50 µg/mL, the correlation coefficient (r^2^) 0.9978, the limit of quantification (LOQ) 1.22 μg/mL, the limit of detection (LOD) 0.40 μg/mL; for chlorogenic acid the calibration range was 10–250 µg/mL, the correlation coefficient (r^2^) 0.9961, the limit of quantification (LOQ) 1.87 μg/mL, the limit of detection (LOD) 0.56 μg/mL; for rosmarinic acid the calibration range was 10–50 µg/mL, the correlation coefficient (r^2^) 0.9968, the limit of quantification (LOQ) 1.61 μg/mL, the limit of detection (LOD) 0.48 μg/mL.

### 3.6. Separation of Unknown Constituent of CIV17-EO 

For the isolation of the essential oil unknown compound, a silica gel chromatographic column (silica gel 60 mesh, particle size: 0.035–0.070 mm, Sigma-Aldrich) was performed. 

An aliquot of 250 μL of CIV17-EO was dissolved in 2 mL of mobile phase: hexane: ethyl acetate (98:2). Precoated silica-gel plates (silica gel 60 F_254_; thickness 0.25 mm; Merck) with the same above mobile phase were used to control the fraction separations: after development, the plate was sprayed with phosphomolybdic acid solution (20% phosphomolybdic acid in EtOH) [40] and heated to 120 °C. The components of the essential oil showed an intense blue color on a yellow background. The solvents of collected fractions were evaporated to dryness with a rotary evaporator (RV 10 digital, IKA^®^-Werke GmbH & CO. KG, Staufen im Breisgau, Germany). 

An aliquot was taken to perform GC-MS to check the purity and another one to perform NMR analysis. The isolation by silica gel column gave 58 mg of the molecule. To assess the purity of the collected fractions, an aliquot was taken and analyzed in the GC-MS: the purity in GC-MS was 94%.

### 3.7. ^1^H-NMR and ^13^C-NMR Analysis

15 mg of essential oil and 5 mg of the pure separated compound were both dissolved in 1 mL of CDCl_3_ and analyzed with a Varian Mercury Plus 400, operating at 400 (^1^H) and 100 MHz (^13^C), respectively.

### 3.8. Antioxidant Activity

The DPPH assay was performed following the method by Cheng et al. [41]: briefly, the DPPH solution was placed on a 96-well plate containing different concentration of extract or pure compounds for 30 min in the dark at room temperature, then the microplates were analyzed with a microplate reader (BioRad, 680 XR, Hercules, CA, USA) and the absorbance was read in triplicate against a blank at 515 nm. The DPPH inhibition in percent was determined by the following formula: IDPPH% = [1 − (A1/A2)] × 100, where A1 was the DPPH absorbance with the extracts and A2 without extracts. Eight different concentrations (range: 0.16–20 μg/mL) of Trolox were prepared and used as positive control. The activity of the extracts was expressed as IC_50_, concentration providing 50% inhibition of the radical. All experiments were performed in triplicate.

The ABTS scavenging activity was evaluated using the method of Horszwald and Andlauer [42]. EO, alcoholic extracts, and pure molecules (rosmarinic acid, eugenol, and Trolox) were tested in a range of concentrations, respectively, 0.58–37 μg/mL, 2.08–133.33 μg/mL, and 0.21–13.33 μg/mL. Aqueous solution (7 mmol/L) of ABTS (10 mL) and 51.4 mmol/L aqueous solution of K_2_S_2_O_4_ (0.5 mL) were mixed to obtain a radical cation solution that has been adjusted spectrophotometrically to 0.7 ± 0.05 at 734 nm. After 6 min of incubation in the dark at room temperature, microplates were analyzed with a microplate reader (BioRad 680 XR), and the absorbance was read at 734 nm in triplicate and against a blank. Antioxidant activity of the samples was expressed as IC_50_, the concentration providing 50% radical inhibition. All experiments were assessed in triplicate and values were reported as mean ± standard deviation.

### 3.9. Cell Lines and Culture Conditions

Adenocarcinomic human alveolar basal epithelial cells (A549) and human keratinocytes (HaCat) were purchased by Istituto Zooprofilattico Sperimentale della Lombardia e dell’Emilia-Romagna (Brescia, Italy) and maintained, respectively, in Ham’s F12 medium and DMEM containing 4.5 g/L and 1 g/L glucose. The cell lines were grown in 75 cm^2^ flasks and cultured in medium supplemented with 10% fetal bovine serum (FBS), 100 U/mL penicillin/streptomycin, and 2 mM L-glutamine in a humidified 5% CO_2_-95% air atmosphere at 37 °C until 80% confluence.

### 3.10. Cell Viability Assay

Cell viability was determined by MTT colorimetric assay [43] as reflected by the activity of succinate dehydrogenase. Briefly, cells were seeded at the density of 2 × 10^4^ cells/well on a 96-well plate. After 24 h, cells were exposed to different concentrations of *H. officinalis* subsp. *aristatus* EO (10–200 µg/mL) and *H. officinalis* subsp. *aristatus* 70% ethanolic extract (10–200 µg/mL) in a final volume of 200 µL of culture medium. Control culture was exposed to vehicle (medium containing 2% FBS) plus DMSO 0.1%. After 72 h of incubation, 20 µL of MTT (5 mg/mL in phosphate-buffered saline, PBS) was added in each well and the plates were incubated for 4 h at 37 °C. The medium was removed and replaced with 100 µL dimethyl sulphoxide to dissolve the formazan crystals. The extent of MTT reduction was measured spectrophotometrically at 570 nm using a microplate reader (BioRad 680 XR).

### 3.11. Characterization of Pharmacokinetic ‘Functional Fingerprint’

Phytochemical pharmacokinetic profiles were explored as described in Selby-Pham et al. [44] with some modifications. In brief, relative abundances (%A) of phytochemicals were sourced from gas chromatography coupled with mass spectrometry (GC-MS). The ‘functional fingerprints’ of phytochemical extracts, describing the relative accumulation of phytochemical abundances against predicted plasma T_max_, were produced as per Selby-Pham et al. using molecular mass and lipophilicity descriptor log P, sourced from the Molinspiration online property calculation toolkit [45]. The phytochemicals clustering according to their pharmacokinetic prevalence can be used as a semi-quantitative tool oriented to extrapolate prevailing, synergistic, or additive effects observed in vitro to in vivo action.

### 3.12. In silico Estimation of Pulmonary Interstitial Concentration

Since the observed partially selective toxic effect on lung cancer cells we tried to evaluate an *in silico* the levels of interstitial and intracellular concentrations in the lung. For this purpose, we used the PK-Sim software, Version 3.0 (Bayer Technology Services GmbH, Leverkusen, Germany). PK-Sim is based on a generic PBPK-model with 17 organs and tissues, including lung. Due to the lack of experimental data, pharmacokinetic properties are sourced from pkCSM online property calculation toolkit, and from ChemAxon online calculator toolkit for the pKa value [46], using graph-based signatures.

### 3.13. Mutagenic Assay

Mutagenicity assay was performed following the plate incorporation method with the histidine-requiring *Salmonella typhimurium* mutant TA98 and TA100 strains purchased by Molecular Toxicology Inc. (Boone, NC, USA; moltox.com). All strains (100 µL per plate of fresh overnight cultures) were checked with and without the addition of 0.5 mL of a 5% S9 exogenous metabolic activator (S9 mix). The lyophilized post-mitochondrial supernatant S9 mix (Aroclor 1254-induced, Sprague–Dawley male rat liver in 0.154 M KCl solution), commonly used for the activation of pro-mutagens to mutagenic metabolites (Molecular Toxicology, Inc., Boone, USA) was stored at −80 °C before use. The concentration tested for all the samples were 5, 10, 20, 50, and 100 µL/plate of a stock solution 50 mg/mL. An amount of 0.5 mL of phosphate buffer or S9 mix for assays with metabolic activation was added to 2 mL molten top agar (0.6% agar, 0.6% NaCl, 0.5 mM L-histidine/biotin solution) at 46 °C, together with 0.1 mL of each sample solution at different concentrations, and 0.1 mL of fully-grown culture of the appropriate tester strain. The ingredients were thoroughly mixed and poured onto minimal glucose agar plates (1.5% agar in 2% Vogel–Bonner medium E with 5% glucose solution). DMSO was used as a negative control (100 µL/plate). Positive controls were prepared as follows: 2-aminoanthracene (2 µg/plate) for both strains with metabolic activation a 2-nitrofluorene (2 µg/plate) and sodium azide (2 µg/plate) for TA98 and TA100 without metabolic activator, respectively. The plates were incubated at 37 °C for 72 h and then the his+ revertant were checked and counted using a 560 Colony Counter (Suntex, New Taipei City, Taiwan). A sample was considered mutagenic when the observed number of colonies was at least twofold over the spontaneous level of revertants. All determinations were made in triplicate.

### 3.14. Statistical Analysis

Data are reported as mean ± standard error of the mean, and “n” was the number of independent experiments performed in triplicate. The statistical analysis for cell viability was calculated using one-way analysis of variance (ANOVA), followed by Dunnett’s Test. The results were considered significant with *p* < 0.01 compared to untreated cells.

## 4. Conclusions

In the last decades, thanks to the increasing number of studies about medicinal plants and their consequent new applications, further attesting their safety and efficacy, the interest of the market for natural products has been awakened. In this context, *H. officinalis* subsp. *aristatus*, already marketed in Europe as an aromatic medicinal plant based on its traditional uses, e.g., as a cooking spice and as a traditional health remedy, evidences however critical issues related to its different chemotypes that must be recognized and standardized in order to attest their efficacy and safety parameters for further guarantee their use by consumers.

The Abruzzo region (Italy), since it reflects a typical Mediterranean plant biodiversity, has been chosen as the study area of *H. officinalis* subsp. *aristatus* with the object to define its phytochemical and pharmaceutical biology profile. Samples from three different areas have been collected during two years and chemically characterized for their secondary metabolite profile. Therefore, the present work presents for the first time, alongside two already known EOs chemotypes, the evidence of a new chemotype never detected before, characterized by the prevalence of limonen-10-yl-acetate (67.9%). Being rich in this compound, which is part of a molecular category under observation by EFSA for its genotoxic potential, this EO was tested to first evaluate in vitro its safety with regard to this criticality. It did not show any mutagenic potential (Ames test), nor cytotoxicity (MTT test) towards the human cell line HaCat. Toxicity towards this cell line, however, was shown by the hydroalcoholic extract, but at a relatively high dose (>100 μg/mL). 

The three samples of *H. officinalis* subsp. *aristatus* showed a different phytochemical composition reflecting different biological effects. The CdG2019-EO was the only one that showed a slight antioxidant activity, while all EtOH extracts, especially the CIV17-UAE one, evidenced interesting antioxidant activity, although with IC_50_ values of an order of magnitude lower than the positive control and the pure molecules identified. Phytocomplexes with antioxidant activity could exert anti-cancer effects through several mechanisms, including alterations in cell signaling, changes in cell cycle progression and modulation of enzymatic activities [23]. Moreover, considering their characteristics, natural compounds could be considered excellent candidates for the development of new intervention strategies to improve the low therapeutic index and the frequent occurrence of chemoresistance of the current anticancer therapies. The evaluation of the biological activity of *H. officinalis* subsp. *aristatus* extracts against human cancer cell line started from these premises, and showed, although in a preliminary way, a statistically significant decrease in cell viability. Although far from the US National Cancer Institute reference values [47], the extracts showed a degree of specificity towards A549 compared to the HaCat cell line, being therefore a starting point for the evaluation of an additional preventive tool for maintaining health status.

Recent in silico pharmacokinetic simulation software provides increasingly accurate tools to evaluate drugs ADME, but the kinetic parameters regulating their reciprocal interactions, as additives, synergistic, or inhibitory effects mediated by hepatic, renal, intestinal metabolism, or drug efflux protein, are so far not available. The implementations of these models will be another challenge for the future.

## Figures and Tables

**Table 1:** Chemical composition of EOs samples of *H. officinalis* subsp. *aristatus;* CIV17-EO (Civitaretenga Essential Oil), NAV19-EO (Navelli Essential Oil) and CdG19-EO (Campo di Giove Essential Oil).

Compound ^a^	CIV17-EO Yield: 0.70% % Area ^b^	NAV19-EO Yield: 0.28% % Area ^b^	CdG19-EO Yield: 0.40% % Area ^b^	exp. AI ^c^
α-pinene	0.3	0.2	0.3	928
sabinene		0.3		965
β-pinene	1.7	3.1	4.3	972
δ-2-carene		0.1		1012
*para*-cymene		1.4		1019
limonene	5.8	0.2	7.6	1024
1,8-cineole	15.5	4.4	39.7	1027
*cis-β*-ocimene	0.7	0.3		1033
*cis*-sabinene hydrate		0.2		1064
*para*-2,4(8)-mentha-diene		0.1		1079
*trans*-sabinene hydrate		0.2		1099
*cis*-thujone		0.3		1103
*trans*-thujone		0.1		1114
dehydrosabina ketone		0.1		1115
*cis*-*para*-menth-2-en-1-ol		0.1		1120
nopinone		0.5		1133
*trans*-sabinol		1.9		1133
*trans*-pinocarveol			0.4	1133
camphor		0.2		1140
*trans*-pinocamphone		11.0		1153
pinocarvone		1.1		1155
*cis*-pinocamphone (or iso-pinocamphone)		43.2		1168
terpinen-4-ol		1.0		1174
*para*-cymen-8-ol		0.2		1185
thuj-3-en-10-al		1.6		1189
myrtenol		1.3		1191
*trans*-carveol		0.1		1217
limonen-10-ol methyl ether	1.9			1235
carvotanacetone		0.2		1243
carvone	0.3	0.2		1244
limonen-10-ol	2.8			1291
perilla alcohol		0.2		1296
β-bourbonene	0.4	0.9	1.0	1381
4,7,10,13,16,19-docosahexaenoic acid, methylester			0.3	1387
methyleugenol		15.8	41.5	1402
(-)-limonen-10-yl-acetate ^d,e^	67.9			1410
germacrene D	1.2			1476
bicyclogermacrene	0.4			1489
elemol			0.4	1547
ledol			1.0	1566
spathulenol	0.4	2.1	0.9	1577
caryophyllene oxide		2.7	1.5	1578
TOTAL IDENTIFIED	99.3	96.1	98.9	

The main compounds are indicated in bold. ^a^ Compounds are listed in order of elution and their nomenclature is in accordance of the NIST (National Institute of Standards and Technology) library. ^b^ Relative peak areas calculated by GC-FID. ^c^ AI exp: arithmetic retention indices calculated on Varian VF-5 ms column using to compare to AI lit: arithmetic retention indices [15]. ^d^ optical rotation determined on isolated compound; ^e^ Structure identification through ^1^H- and ^13^C-NMR.

**Table 2:** The content of phenolic acids and yield of alcoholic extracts of *H. officinalis* subsp. *aristatus*, obtained by Ultrasound Assisted Extraction (UAE), collected in the two years and in three areas: September 2017, Civitaretenga (CIV17-UAE); September 2019, Navelli (NAV19-UAE); October 2019, Campo di Giove (CdG19-UAE).

Ethanolic Extract	Yield	Caftaric acid	Chlorogenic Acid	Rosmarinic Acid
	%	µg of acid/g dried drug ± st. dev.		
**CIV17-UAE**	13.0 ± 0.8	307.53 ± 46.24	7397.38 ± 231.99	759.50 ± 26.14
**NAV19-UAE**	9.33 ± 0.4	285.79 ± 6.29	3684.78 ± 9.04	278.01 ± 8.45
**CdG19-UAE**	7.39 ± 0.3	212.59 ± 0.39	3300.20 ± 42.22	523.96 ± 2.50

**Table 3:** Antioxidant activity of essential oil and hydro-alcoholic extracts of *H. officinalis* subsp. *aristatus* (IC_50_ ± standard deviation and concentration range).

Extracts and Compounds	DPPH IC_50_ (μg/mL)	ABTS IC_50_ (μg/mL)	Concentration Range (μg/mL)
CIV17-EO	/	/	0.156250–10,000.00
NAV19-EO	/	/	0.156250–10,000.00
CdG19-EO	7652.95 ± 478.91	518.90 ± 23.79	0.156250–10,000.00
Thymol	357.56 ± 34.70	12.01 ± 0.73	0.015625–1000.00
CIV17-UAE	39.31 ± 1.53	10.79 ± 1.21	0.781250–100.00
NAV19-UAE	51.71 ± 2.63	20.15 ± 0.57	0.781250–100.00
CdG19-UAE	45.86 ± 3.04	17.19 ± 0.39	0.781250–100.00
Rosmarinic acid	4.31 ± 0.54	1.85 ± 0.03	0.781250–100.00
Chlorogenic acid	7.29 ± 0.17	4.00 ± 0.09	0.781250–100.00
Caftaric acid	6.89 ± 0.94	3.86 ± 0.09	0.781250–100.00
Trolox	5.97 ± 0.22	2.70 ± 0.05	0.781250–0.781250

EO and hydroalcoholic extracts (UAE) of *H. officinalis* subsp. *aristatus* obtained by the sample harvested in: September 2017, Civitaretenga (CIV17); September 2019, Navelli (NAV19); October 2019, Campo di Giove (CdG19).

**Table 4:** Cytotoxic activity of EO and hydro-alcoholic extracts against A549 and HaCat cell line after 72 h of incubation.

	IC_50_ (μg/mL)		Conc. Range (μg/mL)
	A549	HaCat	
CIV17-EO	>200 (87.98 ± 7.82%)	>200 (103.38 ± 7.46%)	10–200
NAV19-EO	>200 (64.25 ± 4.45%)	>200 (100.92 ± 2.55%)	10–200
CdG19-EO	>200 (82.63 ± 6.89%)	>200 (100.76 ± 4.23%)	10–200
CIV17-UAE	>200 (56.14 ± 7.82%)	>200 (59.87 ± 3.32%)	10–200
NAV19-UAE	148.15 ± 5.97	>200 (56.95 ± 2.91%)	10–200
CdG19-UAE	97.30 ± 3.08	>200 (62.17 ± 2.15%)	10–200
Doxorubicin	0.128 ± 0.01	0.130 ± 0.01	0.01–2

In brackets the percentage of cell viability evaluated at the maximum concentration tested (200 μg/mL), when the sample did not reach IC_50_ values.

**Table 5:** Results of Ames Test performed with *H. officinalis* subsp. *aristatus* essential oil obtained from the sample collected on 09/2017 in the Civitaretenga (CIV17-EO).

	CIV17-OE			
	TA98 (rev/C-)		TA 100 (rev/C-)	
conc. %	−S9	+S9	−S9	+S9
5	0.9	0.7	0.8	0.7
10	0.9	1.3	0.7	0.7
20	0.5	1.0	0.9	0.7
50	0.3	1.3	0.1	0.1
100	0.5	0.9	0.1	0.0
C+ (NF)	3.8	4.3	5.6	4.6

## Data Availability

The data presented in this study are available on request from the corresponding author.

## Supplementary Materials

The following is available online at https://www.mdpi.com/article/10.3390/plants10040631/s1, Figure S1: Predicted phytochemical functional fingerprints for *Hyssopus officinalis* subsp. *aristatus* essential oils (CIV17-EO, NAV19-EO and CdG19-EO).
