# Supplementary material for: Wild Italian Hyssopus officinalis subsp. aristatus (Godr.) Nyman: From Morphological and Phytochemical Evidences to Biological Activities
Source: Plants (Basel). 2021 Mar 26;10(4):631. doi: 10.3390/plants10040631 (PMC8065824; doi:10.3390/plants10040631)

Figure S1. Predicted phytochemical "functional fingerprints". Physicochemical properties of phytochemicals present in the three EOs (CIV17-EO, NAV19-EO and CdG19-EO) distilled form *H. officinalis* subsp. *aristatus* (Godr.) Nyman aerial parts were used to predict phytochemical pharmacokinetics. The "functional fingerprints" was produced by plotting the phytochemical abundances against their plasma T_{max} (per PCAP model).

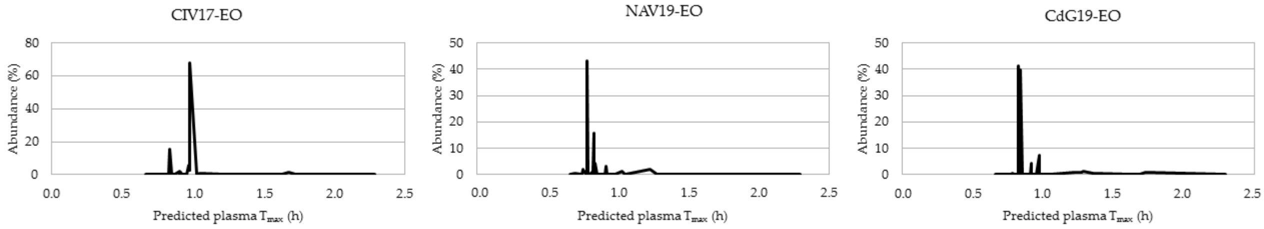

Supplement: Supplementary file 1 [file plants-10-00631-s001.pdf]
